# Improving the Production of Salt-Tolerant Glutaminase by Integrating Multiple Copies of *Mglu* into the Protease and *16S rDNA* Genes of *Bacillus subtilis* 168

**DOI:** 10.3390/molecules24030592

**Published:** 2019-02-07

**Authors:** Xian Zhang, Zhaoyang Xu, Song Liu, Kai Qian, Meijuan Xu, Taowei Yang, Jianzhong Xu, Zhiming Rao

**Affiliations:** 1 State Key Laboratory of Food Science and Technology, Jiangnan University, Wuxi 214122, Jiangsu, China; zx@jiangnan.edu.cn; 2 The Key Laboratory of Industrial Biotechnology of Ministry of Education, School of Biotechnology, Jiangnan University, Wuxi 214122, Jiangsu, China; zhaoyangxv@163.com (Z.X.); 15006180787@163.com (S.L.); xumeijuan@jiangnan.edu.cn (M.X.); ytw1228@163.com (T.Y.); 3 School of Medicine, Yichun University, Yichun 336000, Jiangxi, China; qqkkqian@126.com

**Keywords:** *Bacillus subtilis*, glutaminase, integrated expression, *16S rDNA*

## Abstract

In this study, the *Micrococcus luteus* K-3 glutaminase was successfully over-expressed in the GRAS (Generally Recognized as Safe) *Bacillus subtilis* strain 168 by integration of the *Mglu* gene in the *16S rDNA* locus. This was done in order to screen a strain producing high levels of recombinant glutaminase from the selected candidates. The transcription of the glutaminase genes in the *B. subtilis* 168 chromosome and the expression of glutaminase protein was further assessed by qPCR, SDS-PAGE analysis and an enzyme activity assay. To further increase the production of glutaminase, the *nprB* and *nprE* genes, which encode specific proteases, were disrupted by integration of the *Mglu* gene. After continuous cell culturing without the addition of antibiotics, the integrated recombinant strains showed excellent genetic stability, demonstrating favorable industrialization potential. After the fermentation temperature was optimized, a 5-L bioreactor was used for fed-batch fermentation of the recombinant glutaminase producing strain at 24 °C, and the highest enzyme activity achieved was approximately 357.6 U/mL.

## 1. Introduction

Glutaminase (l-glutamine amidohydrolase, EC 3.5.1.2) catalyzes the hydrolytic degradation of l-glutamine to l-glutamic acid and ammonia. There are two isoforms of glutaminases in mammals, namely, the liver and kidney types, each of which has been purified and characterized [1,2,3]. In microorganisms, glutaminases from *Micrococcus luteus K-3* [4,5], *Aspergillus oryzae RIB40* [6,7], *Bacillus* sp. LKG-01 [8], *Lactobacillus reuteri* KCTC3594 [9] and *Cryptococcus nodaensis* [10] have been characterized. Glutaminase is often used in Chinese traditional fermented foods such as soy source, fermented bean, etc. The main ingredient of umami in traditional fermented seasonings is glutamic acid, which is in the form of glutamine without umami taste in raw materials. Kenji Tomita et al. [11] added pure glutaminase to soy sauce brewing and increased glutamic acid content in the soy sauce by 1.5 times, indicating that glutaminase plays a crucial role during soy sauce fermentation to enrich its flavor characteristics and nutritional value. However, most fermented foods are produced under high NaCl concentrations. That requires that the enzymes added to the fermentation processes must be salt-tolerant. Wakayama et al. [4] showed that the glutaminase from *M. luteus* K-3 was stable and exhibited approximately 1.3-fold higher activity in the presence of 8–16% NaCl than in the absence of NaCl. Therefore, in this study, we synthesized the glutaminase-encoding gene from *M. luteus* K-3 and expressed it in *Bacillus subtilis* 168.

*B. subtilis* is a non-pathogenic bacterial species considered to be Generally Recognized as Safe (GRAS) by the US Food and Drug Administration [12]. Furthermore, *B. subtilis* is used as a probiotic for humans and animals and as an orally administered prophylactic for gastrointestinal disorders, having worldwide commercial applications in the food industry [13,14]. *B. subtilis* is also a food-grade expression host [15] for the expression of proteins and vitamins [16]. As a gram-positive endospore-forming bacteria, *B. subtilis* has several genetic features that distinguish it from the well-known gram-negative bacterium *Escherichia coli* [17]. *B. subtilis* exhibits highly efficient and accurate genetic transformation. Genetic transformation results in the exchange of two homologous DNA segments, one on the chromosome and the other on the plasmid taken up by a competent *B. subtilis* cell [17]. In general, the basic structure of the *B. subtilis* chromosome is unaffected by the presence of heterologous DNA segments, regardless of their size, orientation, or number. Integration of a particular sequence by a Campbell-type mechanism has been frequently used, resulting in tandem repeats of units [18,19,20,21]. However, tandemly repeated homologous sequences have the potential to form deletions, as a result of homologous recombination [22,23,24]. Little is known about the effect of the presence of repetitive sequences in the *B. subtilis* chromosome. Interestingly, if a homologous sequence is present at an unlinked site, it may affect the previously stable genome structure. Increases in the copy number of the particular gene(s) can typically be achieved by generating a stable multi-copy integrant in the rDNA cluster [25,26] and by utilizing multiple integration sites in the *B. subtilis* chromosome.

To make glutaminase better in industrial production, it has been the focus of research so as to screen a salt-tolerant genetically stable glutaminase-producing strain. In this study, a 16S rDNA integration fragment containing an *M. luteus* K-3-derived glutaminase gene (*Mglu*) was obtained by multi-fragment extension, and was transferred into *Bacillus subtilis* to obtain a recombinant strain BSM1. On this basis, two protease-encoding genes *nprB* and *nprE* [27,28] were selected as integration sites and inserted into *Mglu* to eliminate the effect of protease on glutaminase activity. qPCR analysis was used to calculate the copy number and identify the strain with the highest mRNA copy number. Finally, multiple copies of the recombinant strain BSM4 was constructed, enzyme stability analysis showed that the genetic stability of the integrated glutaminase expression construct was better than that of the free plasmid expressing glutaminase. The maximum enzyme activity achieved by fed-batch fermentation in a 5 L bioreactor was 357.6 U/mL. This study provides a reference for the industrial production of glutaminase.

## 2. Results and Discussion

### 2.1. Construction of a Recombinant B. subtilis 168 Strain with Multiple Copies of Integrated Mglu

**Figure 1:**
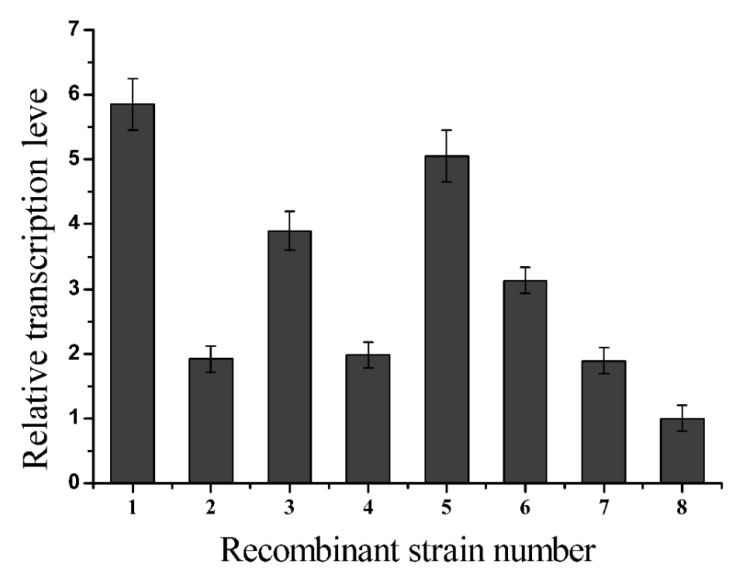
Comparison of the *Mglu* gene transcriptional levels of the transformants with *Mglu* integrated into the *16S rDNA* gene of *B. subtilis*. Transcriptional levels were measured using the $2^{-\Delta \mathrm{tt}}$ assay. The transcriptional level of strain No. 8 was defined as 100%. The standard errors are calculated from three independent biological experiments.

The food-grade expression of glutaminase without the use of an antibiotic resistance gene can be achieved by integrating multiple copies of the *Mglu* gene on the *B. subtilis* genome to overcome the low level of heterogeneous gene expression obtained via single copy integration. The *Mglu* gene was successfully amplified by PCR from the plasmid pUC-*Mglu*, which contains the codon optimized salt-tolerant glutaminase gene from *M. luteus* K-3, according to the *B. subtilis* codon preference. The amplicon was cloned into the vector pMD-18T and was verified by restriction enzyme digestion. Subsequently, the *Mglu* gene was inserted into pMA5 to produce the recombinant plasmid pMA5-*Mglu*, with the insertion verified by DNA sequencing. The sequenced gene had the expected sequence for the codon optimized salt-tolerant glutaminase-encoding gene *M. luteus* K-3. By integrating expression of *Mglu*, the results showed that the *Mglu* gene was successfully inserted into the *16S rDNA* gene of *B. subtilis*, which was screened on for agar plates containing zeocin. Since the *16S rDNA* gene is a multi-copy site in the *B. subtilis* 168 genome, to obtain a recombinant strain with high expression of glutaminase, the obtained positive transformants were further screened. So, we named the eight selected positive transformants as strains 1~8, and real-time PCR was used to determine their transcription level. The analysis of recombinant transcriptional levels was carried out as shown in Figure 1. The results showed that the transcriptional level of strain 1 was the highest among the recombinant strains, suggesting that recombinant strain 1 had the greatest number of copies of the *Mglu* gene in the *B. subtilis* genome. After removing the resistance genes using the Cre recombinase expressed by pTSC, we obtained a recombinant BSM1 strain without a resistance gene, containing multiple copies of the *Mglu* gene in its *16S rDNA* gene. Next, to reduce the degradation of fermentation-derived glutaminase by endogenous protease, we integrated the *Mglu* gene into the *nprB* and *nprE* loci in the BSM1 strain, which code for two different proteases, and obtained the recombinant strains BSM2 and BSM3, respectively. Finally, the strain BSM4 was constructed by inserting the *Mglu* gene into the *nprE* locus of strain BSM2.

### 2.2. L-glutamate Enzyme Activity in the Recombinant Strains

**Figure 2:**
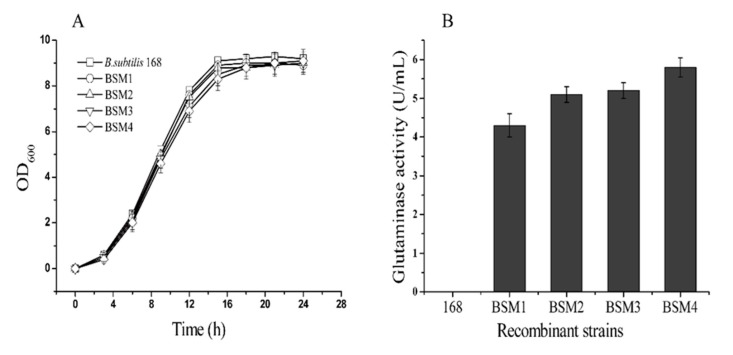
Shake flask fermentation analysis of recombinant strains and *B. subtilis* 168. (**A**) Growth curves of the recombinant and B. subtilis 168 strains. The values shown are the average of three independent measurements and are presented as the error bars; (**B**) the maximum glutaminase activity of the recombinant strains and B. subtilis 168 produced by fermentation. The wild-type B. subtilis 168 strain was used as a negative control. The standard errors are calculated from three independent biological experiments.

To determine the expression level of *Mglu* in the recombinant strains constructed in this study, the *B. subtilis* strains 168, BSM1, BSM2, BSM3, and BSM4 were prepared as described above. As shown in Figure 2A, the growth rate of the recombinant and wild-type *B. subtilis* 168 strains showed the same trend. At approximately 18 h, the glutaminase activity of the BSM1, BSM2, BSM3 and BSM4 strains reached the maximum levels, 4.3, 5.1, 5.2 and 5.8 U/mL (Figure 2B), respectively. However, *B. subtilis* does not have any native glutaminase activity. The enzyme activity of the BSM4 was the highest, which was 35% higher than that of the BSM1 strain. The glutaminase activity of the BSM2 and BSM3 strains improved slightly relative to the BSM1 strain but not to the activity level of the BSM4 strain, possibly due to differences in glutaminase degradation resulting from inactivation of the two proteases. Aoki et al. [29] found that the replication of plasmids in *B. subtilis* was by circular replication and could have a higher copy number. However, replication produced a significant amount of single-stranded DNA, which made the plasmid unstable during replication and easy to lose. It limits the application in industry. The use of integrated expression strategy is to insert a foreign gene into a certain position on the chromosome. The foreign gene replicates with the replication of the host chromosome and can replicate stable. Although the integrated expression level is lower than the plasmid expression, we were able to achieve high levels target protein production by increasing the copy number of the integrated heterologous *Mglu* gene. Furthermore, the protease-mediated degradation of the target protein was decreased by knocking out two *B. subtilis* protease genes.

### 2.3. Purification of Glutaminase

**Figure 3:**
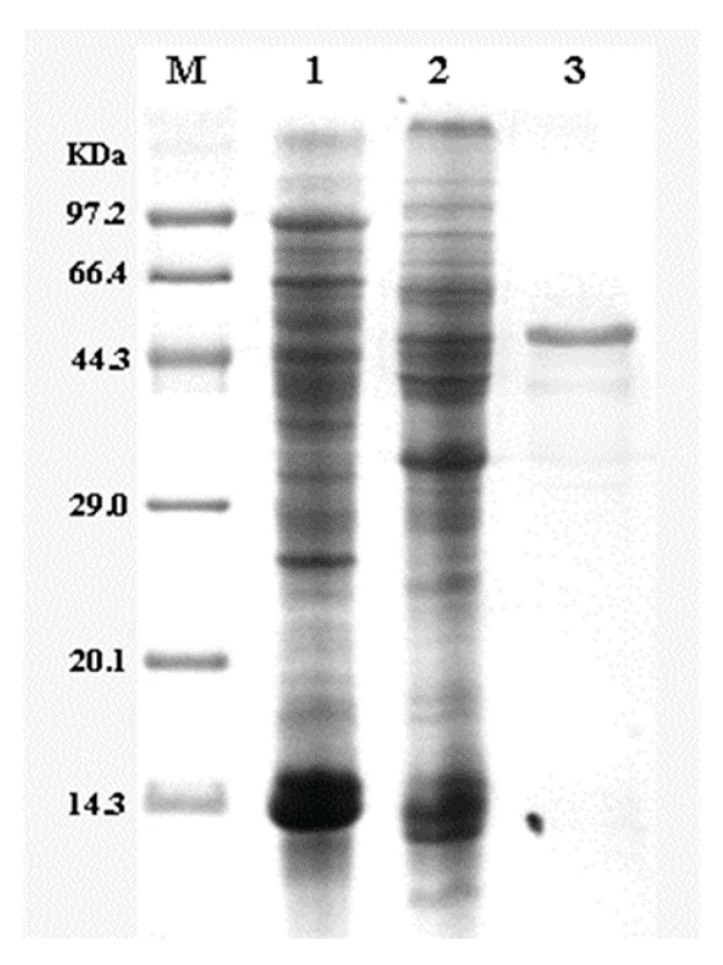
SDS-PAGE analysis of the expression of glutaminase in BSM4. Lane M: protein marker; lane l: cell extract of *B. subtilis* 168 (negative control); lane 2: cell extract of recombinant BSM4; and lane 3: purified glutaminase.

As shown in Figure 3, the Mglu glutaminase was expressed by the BSM4 recombinant strain and produced a visible band at approximately 48 kDa (including the 6×His-Tag), which is the theoretical molecular weight of glutaminase, with *B. subtilis* 168 used as a negative control. The specific activity of purified glutaminase was 1228 U/mg, consistent with previous reports [4]. Kumar et al. [8] reported a glutaminase derived from *Bacillus sp.* LKG-01 (MTCC 10401) with an enzyme activity of 584.2 U/mg, which is much higher than the enzyme from this study. However, since its optimum pH is 11, this is not suitable for industrial production of soy sauce.

### 2.4. Comparison of the Genetic Stability of the Recombinant Strains

**Figure 4:**
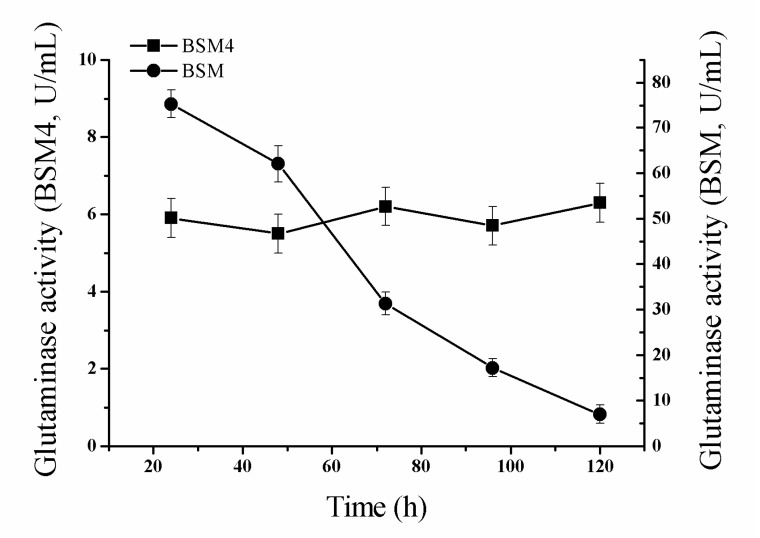
Analysis of the genetic stability of the recombinant strains BSM4 and BSM. The standard errors are calculated from three independent biological experiments.

As mentioned above, integrative expression can increase the genetic stability of foreign genes compared to free plasmids. The results shown in Figure 4 demonstrate that, after continuous transfer of the strains into fresh medium without antibiotic selection, the glutaminase activity of the recombinant strain BSM4 was stable over 48 h, while the glutaminase activity of the recombinant strain BSM (containing free plasmid pMA5-*Mglu*) quickly decreased. These results demonstrated that the genetic stability of the recombinant bacterial strain BSM4 is significantly better than that of BSM in the absence of antibiotic selection. Heterologous over-expression of glutaminase in microorganisms with free plasmids has the obvious advantage that it can increase the enzyme activity greatly. However, the genetic instability of the replicative plasmids has been regarded as the primary problem, which has restricted the industrial application of *B. subtilis* expression system. At the same time, the recombinant strains harboring the resistance gene could not be used to the fermentation of food industry. The integrated expression strain of this study is a green glutaminase expression strain, which does not carry the resistance gene. Therefore, BGM4 is more suitable for industrial production than strains using a replicative plasmid system.

### 2.5. 5-L Bioreactor Fermentation Analysis of BSM4

The results of glutaminase production during fermentation at 30 °C without feed medium were presented in Figure 2B. The glutaminase production increased sharply as the BSM4 recombinant strain grew into the logarithmic growth phase. However, after the logarithmic growth phase, the glutaminase activity decreased significantly. The salt-tolerant glutaminase was not stable at 35 °C after 10 min [7,30]. These results indicated that fermentation at 30 °C was not optimal for glutaminase production and that the fermentation temperature should be further optimized.

**Figure 5:**
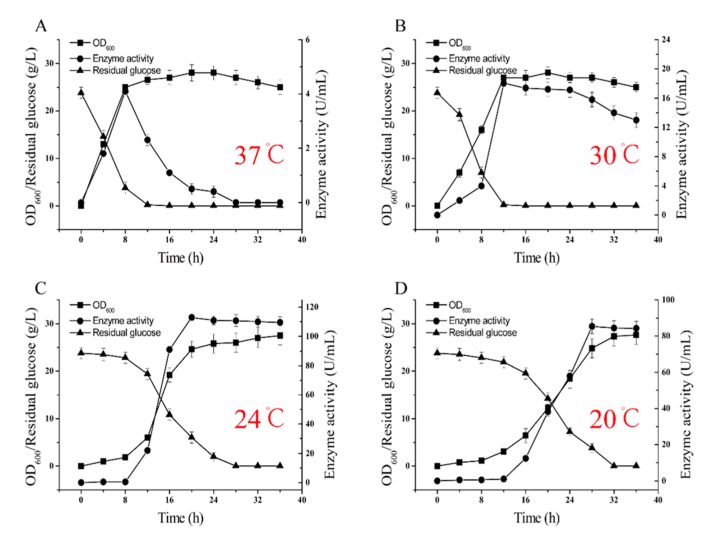
The effect of temperature on the fermentation of glutaminase. (**A**) Glutaminase fermentation at 37 °C; (**B**) glutaminase fermentation at 30 °C; (**C**) glutaminase fermentation at 24 °C; and (**D**) glutaminase fermentation at 20 °C. The standard errors are calculated from three independent biological experiments.

Therefore, the effects of fermentation temperature (37 °C, 24 °C, and 20 °C) on glutaminase fermentation were investigated. Interestingly, the fermentation temperature had a significant effect on cell growth and glutaminase production. Changes in the OD_600_, glutaminase activity, and the residual glucose concentration are shown in Figure 5A–D. The highest glutaminase activity (115.2 U/mL) was obtained with a fermentation temperature of 24 °C, significantly higher than that observed by fermenting at 37 °C (4.4 U/mL), 30 °C (18.3 U/mL), or 20 °C (86.1 U/mL). In addition, we observed that the maximum biomass obtained at different fermentation temperatures was approximately the same. This result indicates that the fermentation temperature of 24 °C is more suitable for producing glutamine.

**Figure 6:**
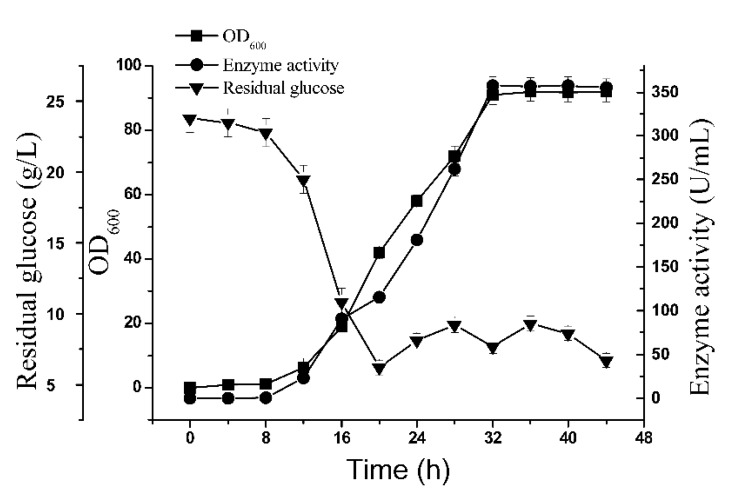
Fed-batch fermentation of the recombinant strain BSM4. The standard errors are calculated from three independent biological experiments.

Thus, we concluded that glutaminase biosynthesis in BSM4 was partly associated (Figure 5A–D) with a higher biomass and enhanced glutaminase biosynthesis since biomass production of the recombinant strain BSM4 was restricted by the nutrient material without feed medium. Therefore, we chose a 5-L bioreactor for the glutaminase fermentation, using the fed-batch strategy to obtain a higher production of glutaminase. This approach was used to prevent aerobic metabolism in order to strengthen cell growth and product formation while decreasing organic acid production by keeping the glucose concentration at low levels [31]. Thus, glucose was maintained at 5–10 g/L through the fed-batch operational strategy. As shown in Figure 6, a higher biomass yield (OD_600_ = 92) and glutaminase activity (357.6 U/mL), which was higher than that observed for *B. subtilis* RSP-GLU [32,33] and *Stenotrophomonas maltophilia* NYW-81 [34], was obtained by applying the fed-batch strategy with a fermentation temperature of 24 °C. The application of the fed-batch strategy had a positive influence on the enhancement of the glutaminase activity. However, the enzyme activity gradually decreased after 32 h, possibly due to enzyme degradation. To the best of our knowledge, this is the highest production of glutaminase ever reported by a strain containing multiple chromosomally integrated glutaminase genes.

## 3. Materials and Methods

### 3.1. Gene Source and Materials

The salt-tolerant glutaminase gene sequence from *M. luteus* K-3 in the NCBI database (Accession number: DQ019448.1) was designed and synthesized according to the preference of *B. subtilis* (the codon optimization result is shown in Figure S1 in the Supplementary Materials) by Sangon Biotech., Ltd. (Shanghai, China). PCR reagents, T4 DNA ligase, and restriction enzymes were purchased from TaKaRa (Dalian, China), and RNAiso Plus, the PrimeScript RT Reagent Kit and ChamQ^TM^SYBR^®^qPCR Master Mix were purchased from Vazyme (Nanjing, China). Mini Plasmid Rapid Isolation and Mini DNA Rapid Purification Kits were purchased from Sangon Biotech (Shanghai, China). Experiments were performed in accordance with the manufacturer’s instructions.

### 3.2. Strains, Plasmids, and Primers

The strains, plasmids, and primers used in this study are listed in Table S1.

### 3.3. PCR Amplification of DNA Fragments

PCR was performed using a Peltier Thermal Cycler (Bio-Rad, Hercules, CA, USA). The PCR mixture (50 µL) contained 1 ng of template, 200 µM dNTPs, 20 µM primers, and 1 U PrimeSTAR HF DNA Polymerase. The amplification conditions were as follows: one cycle at 98 °C for 180 s, followed by 28 cycles of denaturation at 98 °C for 10 s, annealing at 61 °C for 15 s, and extension at 72 °C for 150 s. The amplicons were separated by electrophoresis in a 0.8% agarose gel, after which the PCR products were excised and gel-purified.

### 3.4. Construction of the Recombinant Plasmid and Integrated Fragments

**Figure 7:**
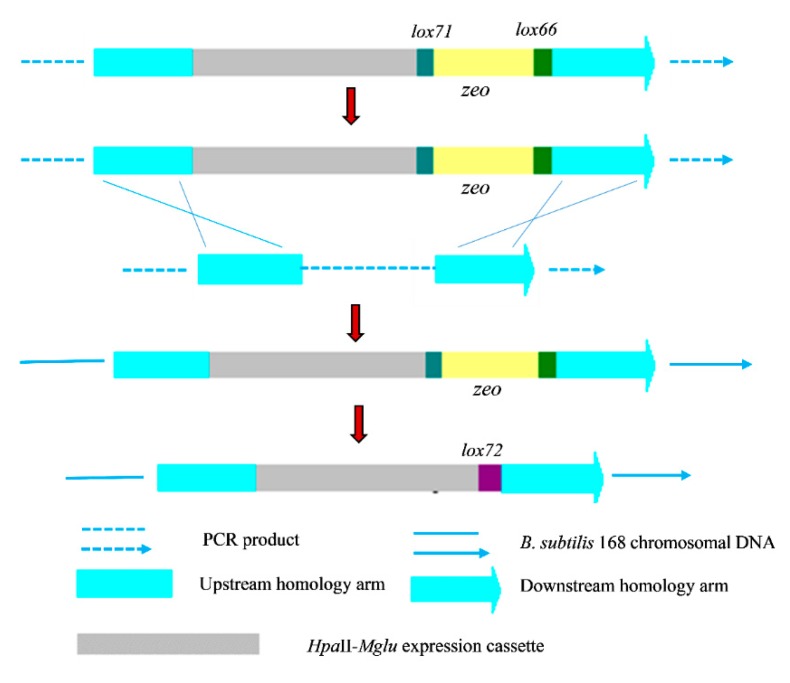
Strategy for integrating the *Mglu* gene in the *B. subtilis* 168 chromosome. An integrative fragment contained the *lox71*-*zeo*-*lox66* cassette with two regions having homology to the *B. subtilis* 16 chromosome and the *Hpa*II-*Mglu* cassette. Due to the homologous regions that flank the *lox71*-*zeo*-*lox66* cassette and the *Hpa*II-*Mglu* cassette, the corresponding gene in the *B. subtilis* 168 chromosome DNA can be disrupted, and the *zeo* gene and *Hpa*II-*Mglu* cassette is introduced. Next, the *zeo* gene was deleted through recombination between the *lox71* and *lox66* sites using the Cre/lox system, yielding the *lox72* site.

The *Mglu* gene was amplified using the primers P1 and P2 and pUC-*Mglu* as a template. The plasmid pMA5-*Mglu* was constructed by inserting *Mglu* between the *Nde*I and *Bam*HI sites of pMA5. PCR products were fused as described by Shevchuk, et al. [35]. Two primer pairs, P3/P4 and P9/P10, were used to amplify the *16S rDNA* arm fragments using *B. subtilis* genomic DNA as a template. The *lox71*-*zeo*-*lox66* cassette was amplified from the p7Z6 vector using the P7/P8 primer pair, and the *Hpa*II-*Mglu* expression cassette was amplified from pMA5-*Mglu* using the primers P5 and P6. Finally, the four DNA fragments were fused to completely integrate the fragment via PCR using PrimeSTAR GXL DNA Polymerase [35]. The flow chart of the construction of the integrated expression is shown in Figure 7. The *nprE* and *nprB* PCR products were fused following the same procedure, as above, using the primers P11-P18 and P19-P26.

### 3.5. Preparation of RNA and cDNA Synthesis

Total RNA was extracted from *B. subtilis* cells using RNAiso Plus, and cDNA was synthesized with a PrimeScript RT reagent kit.

### 3.6. Analysis of the Relative Transcription Levels of Recombinant Glutaminase

qPCR was performed with a StepOne^TM^ Touch qPCR system using ChamQ™ SYBR^®^ qPCR Master Mix. The PCR mixture was prepared containing 0.4 µL of *B. subtilis* DNA (1:100 diluted), 10 µL of 2× ChamQ™ SYBR^®^ qPCR Master Mix, 0.4 µL of each primer (10 µM), 0.4 µL 50× ROX Reference Dye 1, and ddH_2_O to a total volume of 20 µL. The *16S rDNA* and *Mglu* gene primers were P27-P30, as shown in Table S1. Thermal cycling conditions consisted of one cycle of 9 5 °C for 30 s, followed by 40 cycles of 95 °C for 10 s and 60 °C for 30 s. Melting curves were analyzed from 60 to 95 °C to ensure that primer-dimers or nonspecific products did not form during the PCR. Negative controls without a template were also included to detect any spurious signals indicating DNA contamination. All reactions were performed in triplicate. To standardize the results, the relative abundance of the *16S rDNA* gene was used as an internal standard [36].

### 3.7. Transformation and Selection of the Recombinant Strains

The ligated pMA5-*Mglu* plasmid was transformed into *E. coli* JM109. Positive colonies were selected on agar plates containing 50 mg/L ampicillin, and the plasmids were confirmed using restriction enzyme analysis and DNA sequencing. The confirmed recombinant plasmids pMA5-*Mglu* were then transformed into *B. subtilis* 168 [37]. The transformants were screened for their ability to grow on Luria-Bertani (LB) medium (10 g/L tryptone, 5 g/L yeast extract and 10 g/L NaCl) agar plates containing 100 mg/L kanamycin. The *16S rDNA* site integration fragments were introduced into *B. subtilis* 168 competent cells, and the transformants were selected for on agar plates containing 25 mg/L zeocin. Transcriptional levels were analyzed to identify recombinant strains with high levels of *Mglu* gene transcription. The competent cells were transformed with the plasmid pSTC, which expresses the Cre recombinase catalyzing the recombination of DNA between *loxP* sequences [38] and removes the *zeo* resistance marker. Next, the temperature-sensitive plasmid pSTC was eliminated by increasing the culture temperature to 51 °C. The *nprB*- and *nprE*- inactivated strains were constructed following the procedure described above. The strains used in this work are shown in Table S1.

### 3.8. Enzyme Activity Assay

Glutaminase activity was determined by measuring the formation of l-glutamate. The reaction mixture (1 mL) contained 440 μL of 100 mM glutamine, 440 μL of 100 mM Tris-HCl buffer (pH 7.5), and 20 μL of crude glutaminase. The reaction mixture was incubated at 37°C for 5 min and was terminated by the addition of 100 μL of 15% (*w*/*v*) trichloroacetic acid. After filtration with a 0.22-µm filter, the glutamic acid in the supernatant was analyzed through HPLC. However, a biosensor analyzer (Biology Institute of Shandong Academy of Science) can be used to determine the glutamic acid content more quickly and efficiently by injection the sample to the machine without any pretreatment. As shown in Table S2 and Figure S2, the results of the enzyme activity assays using the biosensor analyzer and HPLC methods were consistent. Thus, the l-glutamate contents were both measured using the HPLC method and a biosensor analyzer in this study. One unit of l-glutaminase was defined as the amount of enzyme required to produce 1 μmol of glutamic acid per minute under the assay conditions. The protein concentration was determined by the Bradford method with bovine serum albumin used as the standard.

### 3.9. Preliminary Determination of Enzyme Activity

The recombinant strains were cultured on LB agar plates for 12 h, and the resulting colonies were inoculated into 10 mL LB medium and cultured for 12 h at 37 °C. Next, 1 mL of the culture was transferred into a 1000-mL flask containing 100 mL of fermentation medium (25 g/L glucose, 40 g/L yeast extract, 4 g/L NH_4_Cl, 1.875 g/L K_2_HPO_4_·3H_2_O, 1.125 g/L KH_2_PO_4_, 1.845 g/L MgSO_4_, and 2 g/L C_5_H_8_NO_4_Na·H_2_O) and was cultured at 30 °C for 24 h. Cells were harvested by centrifugation at 10,000× *g* for 5 min at 4 °C and washed twice with 50 mM Tris-HCl (pH 7.5). The precipitate was resuspended in 5 mL of 50 mM Tris-HCl (pH 7.5), after which 50 μL of lysozyme (200 g/mL) was added, and the mixture was incubated 90 min at 4 °C. The cell paste was then sonicated for 10 min (for 1 s at 2-s intervals). Finally, cell extracts were centrifuged for 25 min at 10,000× *g* to remove cell debris and the crude enzyme obtained from the supernatant was filtered through a 0.45-µm filter and used to measure the crude glutaminase activity.

### 3.10. Purification and SDS-PAGE Analysis of Glutaminase

The recombinant strain BSM4 was cultivated in fermentation medium at 30 °C for 24 h, and the crude enzyme was obtained as described above. The N-terminal His-tagged glutaminase was purified in a single step using Ni-NTA affinity chromatography, followed by multiple washes, and then the protein was eluted according to the manufacturer’s protocol (GE Healthcare Bio-Sciences). The purified glutaminase and crude enzyme fractions were analyzed using sodium dodecyl sulfate-polyacrylamide gel electrophoresis (SDS-PAGE) analysis (12% acrylamide).

### 3.11. Enzyme Stability of the Recombinant Strains

The recombinant strains BSM4 and BSM were cultured on LB agar plates for 12 h, and the resulting colonies were used to inoculate 10 mL of LB medium, which was cultured for 12 h at 37 °C. The enzyme stability was assessed by transferring 500 μL of the cultures into 500-mL flasks containing 50 mL of fermentation medium without any added antibiotics. After culturing for 24 h at 30°C, 500 μL of each culture was transferred into new flasks containing 50 mL of fresh fermentation medium without antibiotics, and the enzyme activities of the recombinant strains were measured. The cultures were transferred to fresh media without antibiotics every 24 h, with simultaneous measurements of enzyme activity [20].

### 3.12. Glutaminase Fermentation in a 5-L Bioreactor

The recombinant bacterial strain BSM4 was grown on LB agar plates and used to inoculate a seed culture that was grown for 12 h at 37 °C in LB medium. Next, the seed culture was used to inoculate 100 mL of LB medium (diluted to 1% (*v*/*v*)) in a 1000-mL flask. The culture was grown at 37 °C and 180 rpm and then was transferred into a 5-L stirred fermenter (BIOTECH-5BG, BAOXING Co., Shanghai, China) containing 2 L of fermentation medium. The agitation speed was controlled at 450 rpm, with the pH maintained at 7.0 by the automatic addition of a 30% ammonia solution and a 30% phosphate solution. The medium (containing 500 g/L glucose and 500 g/L yeast extract) was fed into the fermenter when the remaining glucose concentration was below 10 g/L. The air flow rate was maintained at 4 L/min, and the glucose concentration in the medium (5–10 g/L) was controlled by the addition of feed medium.

## 4. Conclusions

*B. subtilis* is a bacterial species considered as GRAS that is widely used in the food industry. Compared with a recombinant *E. coli* strain expressing a *M. luteus* K-3 glutaminase from a plasmid, the food-grade recombinant *B. subtilis* constructed in this study was safer. Although the expression level of genes was a barrier for integrative expression, we achieved a high production of salt-tolerant glutaminase by increasing the copy number of the heterologous gene. The specific enzyme activity increased slightly as the sites expressing the heterogeneous gene increased, reaching a maximum with the BSM4 recombinant strain, which had the most integrated *Mglu* expression sites. By comparing enzyme activity results, we could preliminary estimate that there are about 5 or 6 Mglu gene copies in BSM1, and thus 7 or 8 copies in BSN4. The molecular weight of glutaminase from the BSM4 recombinant strain was consistent with that which has been previously reported. In addition, the maximum enzyme activity of the recombinant strain BSM4 was 357.6 U/mL, which was obtained by fermentation with the feeding of glucose and yeast extract in a 5-L bioreactor. The recombinant BSM4 constructed in this study is promising for the industrial production of glutaminase. However, despite the highest production of glutaminase achieved by this work, glutaminase stability remains an obstacle for scaling-up its fermentation. Protein engineering methods such as site-directed mutagenesis and directed evolution can help to increase the relative activity, pH stability and thermostability of the enzyme, and could guide to searching a comprehensive mutant with better salt-tolerant ability or lower pH optimum that favorable to fermented food to improve its potential for industry application. In addition, expression regulatory methods such as displaying proteins on the surface of spores, optimization of the promoters and RBS sites have been widely used in engineered recombinants for increasing the yield of target proteins. These will be effective means for our future research.

## Supplementary Materials

Supplementary materials are available online. Figure S1. The result of the codon optimization of salt-tolerant glutaminase from *Micrococcus luteus* K-3 according to the preference of *Bacillus subtilis*. Figure S2. HPLC analysis of l-glutamine and l-glutamic acid. Table S1. Bacterial strains, plasmids and primers used in this study. Table S2. Glutamate analysis in the reaction mixture via HPLC and biosensor analyzer analyses.
