# Supplementary material for: Improving the Production of Salt-Tolerant Glutaminase by Integrating Multiple Copies of Mglu into the Protease and 16S rDNA Genes of Bacillus subtilis 168
Source: Molecules. 2019 Feb 7;24(3):592. doi: 10.3390/molecules24030592 (PMC6384544; doi:10.3390/molecules24030592)
Supplement: Supplementary file 1 [file molecules-24-00592-s001.pdf]

Improving the production of salt-tolerant glutaminase by integrating multiple copies of *Mglu* into the protease and *16S rDNA* genes of *Bacillus subtilis* 168

Xian Zhang[1, 2], Zhaoyang Xu[2], Song Liu[2]，Kai Qian[3], Meijuan Xu[2], Taowei Yang[2], Jianzhong Xu[2*], Zhiming Rao[1, 2*]

[*]**Corresponding author**: raozhm@jiangnan.edu.cn (Z.R.); xujianzhong@jiangnan.edu.cn (J. X.)

[1]State Key Laboratory of Food Science and Technology，Jiangnan University, Wuxi 214122, Jiangsu, People's Republic of China

[2]The Key Laboratory of Industrial Biotechnology of Ministry of Education, School of Biotechnology, Jiangnan University, Wuxi 214122, Jiangsu, People's Republic of China

[3]School of Medicine, Yichun University, Yichun 336000, Jiangxi, People's Republic of China

ATGCGTCACCCTATCCCAGACTACCTGGCCAGCCTGGTAACCGAGCTGGGTGCAGTAAACC
CTGGCGAAACCGCTCAGTACATCCCGGTGCTGGCAGAGGCAGATCCAGACCGTTTCGGTAT
CGCTCTGGCTACCCCGACTGGTCGTCTGCATTGCGCAGGTGACGCTGATGTGGAGTTCACC
ATTCAGTCCGCGTCCAAACCGTTCACCTACGCGGCTGCGCTGGTCGACCGTGGTTTCGCAG
CTGTGGACCGTCAGGTAGGTCTGAACCCGAGCGGTGAGGCTTTCAACGAGCTGAGCCTGG
AGGCAGAAAGCCACCGTCCGGACAACGCAATGATCAACGCGGGTGCACTGGCTGTACACC
AGCTGCTGGTCGGTCCGGAAGCATCTCGTAAGGAACGTCTGGACCGTGCAGTGGAAATCAT
GTCCCTGCTGGCCGGTCGTCGTCTGTCCGTGGATTGGGAAACGTACGAATCCGAAATGGCG
GTCAGCGACCGCAACCTGTCCCTGGCGCACATGCTGCGTAGCTATGGCGTGCTGCAGGACT
CCGCAGAAGAAATCGTGGCCGGCTACGTGGCACAGTGCGCAGTCCTGGTCACTGTCAAAG
ACCTGGCGGTGATGGGCGCATGTCTGGCAACCGGTGGTATCCACCCGATGACGGGTGAACG
TATGCTGCCGTCTATCGTGGCGCGTCGTGTGGTGTCTGTTATGACCTCCTCTGGCATGTATGA
CGCGGCCGGCCAGTGGCTGGCTGATGTAGGCATCCCGGCTAAATCTGGTGTTGCGGGCGGT
GTTCTGGGTGCTCTGCCGGGTCGTGTTGGTATCGGTGTTTTCAGCCCGCGCCTGGATGAAGT
TGGCAACTCTGCGCGTGGCGTTCTGGCTTGTCGTCGCCTGTCTGAAGACTTCCGCCTGCAT
CTGATGGACGGCGACTCTCTGGGTGGTACCGCTGTTCGTTTTGTTGAACGCGAAGGCGACC
GCGTTTTTCTGCACCTGCAGGGCGTTATCCGCTTTGGCGGCGCGGAAGCGGTTCTGGACGC
TCTGACCGATCTGCGTACGGGTGCTGAGAAACCGGGTACTGGCTGGGATGCTGCTGTTTAT
CCGCGCTGGCAAGAAGCCGCCGCCGATCGTGCGGCTCTGTCTGCGGCGACTGGCGGCGGT
GCCGTTCATGAAGCGGCAGCCGCTGCGGCGCGTGATGAGAATGATGGCCCAATTCGTACTG
TTGTTCTGAATCTGGCCCGTGTTGATCGTATTGATGACGTAGGTCGCCGCCTGATTGCCGAA
GGCGTTCGCCGTCTGCAAGCGGATGGCGTACGCGTAGAAGTAGAAGATCCGGAACGCATTC
TGCCGCTGGAAGAAGCGGGCGCGCACTAA

Figure S1. The result of the codon optimization of salt-tolerant glutaminase from *Micrococcus luteus* K-3 according to the preference of *Bacillus subtilis.*

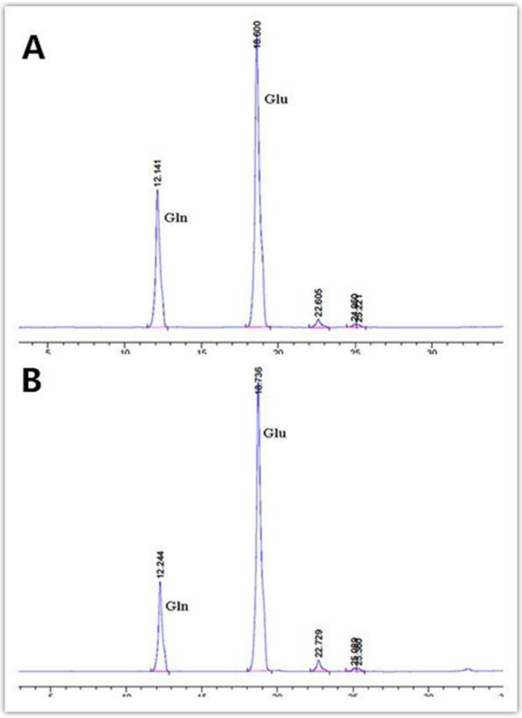

Figure S2. HPLC analysis of L-glutamine and L-glutamic acid. a) HPLC analysis of L-glutamine and L-glutamic acid (0.5 g/L); b) HPLC analysis of the reaction mixture (dilution factor=10).

Table S1. Bacterial strains, plasmids and primers used in this study.

Strain/plasmid/primer	Properties	Source
**Strains**		
E. coli JM109	*recA1, endA1, gyrA96, thi-1, hsd R17* (r_k^- m_k^+) *supE44*	Invitrogen
B. subtilis 168	*trpC2*	Laboratory stock
BSM	*B. subtilis* 168 containing pMA5-*Mglu* (Km^R)	This work
BSM1	*B. subtilis* 168 containing *Mglu* integrated in 16S rDNA site	This work
BSM2	*B. subtilis* 168 containing *Mglu* integrated in 16S rDNA site and *nprB* site.	This work
BSM3	*B. subtilis* 168 containing *Mglu* integrated in 16S rDNA site and *nprE* site.	This work
BSM4	*B. subtilis* 168 containing *Mglu* integrated in 16S rDNA site, *nprB* site and *nprE* site	This work
**Plasmids**		
pMD-18T	Amp^R; MCS	Takara
pMA5	*E. coli* (Amp^R); *B. subtilis* (Km^R); MCS	Laboratory stock
pUC-*Mglu*	*E. coli* (Amp^R); *Mglu*	Sangon Biotech
pMA5-*Mglu*	*E. coli* (Amp^R); *B. subtilis* (Km^R);*Mglu*	Laboratory stock
P7Z6	pMD18-T ligated with lox71-*zeo*-lox66 cassette	[34]
pSTC	*E. coli* (Amp^R); *B. subtilis* (Km^R) temperature sensitive in *B. subtilis*; containing $P_{spac\text{-}cre}$ expression cassette	[34]
**Primers**		
P1	ACCGGGATTCATGCGTCACCCTATCCCAGACTACC(*NdeI*)	
P2	ACCGCATATGTTAGTGGTGGTGGTGGTGGTGGTGCGCGCCCGCTTCTT(*Bam HI*)	
P3	GCTGGCGGCGTGCCTAATACATGCAAGTCGAGCGGACAGATGGGAGCTT	
P4	ATTTTTATTTTGTCCGTTTTGTCTAGCTTATGGAATTCCACTCTCCTCTT	
P5	TTGAGTGCAGAAGAGGAGAGTGGAATTCCATAAGCTAGACAAAACGGACA	
P6	GGGTACCGAGCTCGAATTCGTAATCATGGTGCTAGCTTGAGCTCGACTCT	
P7	CTAATCCTCTAGAGTCGAGCTCAAGCTAGCACCATGATTACGAATTCGAG	
P8	CCACATCTCTACGCATTTCACCGCTA	

	CACGATTCTACCGTTCGTATAATG
P9	GTATAGCATACATTATACGAACGGTA
	GAATCGTGTAGCGGTGAAATGCGT
P10	ACCTTCCGATACGGCTACCTTGTTAC
	GACTTCACCCCAATCATCTGTCCCA
P11	GTGGGTTTAGGTAAGAAATTGTCTGT
	TGCTGTCGCTGCTTCGTTTATGAG
P12	ATTTTTATTTTGTCCGTTTTGTCTAGC
	TTATTTTGCAAATCATATGTGAT
P13	CACCCAAATCATCACATATGATTTGC
	AAAATAAGCTAGACAAAACGGACA
P14	GGGTACCGAGCTCGAATTCGTAATCA
	TGGTGCTAGCTTGAGCTCGACTCT
P15	CTAATCCTCTAGAGTCGAGCTCAAGC
	TAGCACCATGATTACGAATTCGAG
P16	ACAAGCGTGCCCGGAAGGCGGCTTT
	GTCTGGTGCCAAGCTTGCATGCCTG
P17	CGTCGACCTGCAGGCATGCAAGCTT
	GGCACCAGACAAAGCCGCCTTCCGG
P18	TTACAATCCAACAGCATTCCAGGCTG
	CTTCAACTTTAGCGGCATCAGT
P19	TTGCGCAACTTGACCAAGACATCTCT
	ATTACTGGCCGGCTTATGCACAGC
P20	ATTTTTATTTTGTCCGTTTTGTCTAGC
	TTATTCAGGTTCTTCGCGTCAAA
P21	GATCAATACATTTGACGCGAAGAAC
	CTGAATAAGCTAGACAAAACGGACA
P22	GGGTACCGAGCTCGAATTCGTAATCA
	TGGTGCTAGCTTGAGCTCGACTCT
P23	CTAATCCTCTAGAGTCGAGCTCAAGC
	TAGCACCATGATTACGAATTCGAG
P24	CGATCAGTTGAGACAAAAGCGTAAA
	CAAGGGTGCCAAGCTTGCATGCCTG
P25	CGTCGACCTGCAGGCATGCAAGCTT
	GGCACCCTTGTTTACGCTTTTGTCT
P26	TGTTTGCTCATAGAATGCCGACAGCC
	TCATACGCCTTTTCGACTGAAGCT
P27	GCAGCAGTAGGGAATCTT
P28	GTGGCTTTCTGGTTAGGT
P29	GGACTCCGCAGAAGAAAT
P30	CATAACAGACACCACACG

Amp^R Ampicillin resistant, Km^R kanamycin resistant. The highlighted text in the primer sequences represent restriction enzyme sites.

Table S2. Glutamate analysis in the reaction mixture via HPLC and biosensor analyzer analyses.

	I-1	I-2	II-1	II-2
HPLC analysis (g/L)	2.7729	2.8066	3.3910	3.2418
Variance	0.0584	0.0875	0.0613	0.0450
Glutamate glucose analyzer (g/L)	2.7000	2.7333	3.2667	3.2667
Variance	0.1000	0.0577	0.0577	0.0577
Relative result (%)	97.3717	97.3887	96.3324	100.7673

I and II represented two independent samples.
